# Comparative effect of *Piper betle*, *Chlorella vulgaris* and tocotrienol-rich fraction on antioxidant enzymes activity in cellular ageing of human diploid fibroblasts

**DOI:** 10.1186/1472-6882-13-210

**Published:** 2013-08-16

**Authors:** Suzana Makpol, Thong Wei Yeoh, Farah Adilah Che Ruslam, Khaizurin Tajul Arifin, Yasmin Anum Mohd Yusof

**Affiliations:** 1 Department of Biochemistry, Faculty of Medicine, Universiti Kebangsaan Malaysia, Jalan Raja Muda Abdul Aziz, Kuala Lumpur 50300, Malaysia

**Keywords:** Antioxidant, *Piper betle*, *Chlorella vulgaris*, Tocotrienol-rich fraction (TRF), Cellular ageing, Fibroblasts

## Abstract

**Background:**

Human diploid fibroblasts (HDFs) undergo a limited number of cellular divisions in culture and progressively reach a state of irreversible growth arrest, a process termed cellular ageing. Even though beneficial effects of *Piper betle*, *Chlorella vulgaris* and tocotrienol-rich fraction (TRF) have been reported, ongoing studies in relation to ageing is of interest to determine possible protective effects that may reverse the effect of ageing. The aim of this study was to evaluate the effect of *P. betle*, *C. vulgaris* and TRF in preventing cellular ageing of HDFs by determining the activity of antioxidant enzymes viz.; catalase, superoxide dismutase (SOD) and glutathione peroxidase.

**Methods:**

Different passages of HDFs were treated with *P. betle*, *C. vulgaris* and TRF for 24 h prior to enzymes activity determination. Senescence-associated beta-galactosidase (SA β-gal) expression was assayed to validate cellular ageing.

**Results:**

In cellular ageing of HDFs, catalase and glutathione peroxidase activities were reduced, but SOD activity was heightened during pre-senescence. *P. betle* exhibited the strongest antioxidant activity by reducing SA β-gal expression, catalase activities in all age groups, and SOD activity. TRF exhibited a strong antioxidant activity by reducing SA β-gal expression, and SOD activity in senescent HDFs. *C. vulgaris* extract managed to reduce SOD activity in senescent HDFs.

**Conclusion:**

*P. betle, C. vulgaris*, and TRF have the potential as anti-ageing entities which compensated the role of antioxidant enzymes in cellular ageing of HDFs.

## Background

Ageing is a multi factorial process which involves progressive decline in body function and finally leads to death [1]. Cellular ageing or replicative senescence is a condition where the cell cycle arrest occurs permanently [2], when the cell fails to initiate DNA synthesis and transition from G1 to S phase of the cell cycle [3]. Studies on cellular senescence normally use fibroblast cell culture system which has a limited replicative ability. Senescent cells exhibit morphological changes such as cellular enlargement, loss of contact inhibition and become less polar [4]. Senescence-associated β-galactosidase is one of the markers widely used to indicate replicative senescence since its activity increases with passage of time [5].

According to Harman’s hypothesis (1956), cells undergo ageing due to oxidative stress, which is caused by the presence of free radicals [6]. Utilization of oxygen molecule during normal metabolism within the cell is the main source of free radicals [7]. When an overproduction of free radicals and reactive oxygen species exceeds the capacity of antioxidant mechanism of the cells, the cells will experience oxidative stress [8]. Oxidative stress in tissues has been linked to ageing and degenerative diseases [9].

Many biological functions decline in ageing due to excessive production of reactive oxygen species over the antioxidant protective mechanism [10]. Previous study reported that the half life of an organism depends on the effectiveness to overcome the oxidative environment [11].

Aerobic organisms have their antioxidant defence mechanism which protects against oxidative stress such as the presence of antioxidant enzymes; superoxide dismutase, catalase and glutathione peroxidase. Superoxide dismutase catalyzes the conversion of superoxide anion to oxygen and hydrogen peroxide [12]. Catalase converts hydrogen peroxide to water and oxygen [8,13] while glutathione peroxidase reduces hydrogen peroxide by oxidizing GSH (reduced glutathione) [8].

*Piper betle* (also identified as betel) Linn., locally known as *sirih* is a semi woody plant under family *Piperaceae*[14]. In many Asian countries, the leaves of the *P. betle* are used in masticatory for recreational and medicinal purposes [15]. The leaves of *P. betle* act as a potential source of natural antioxidants [9]. The antioxidant activity can be attributed to the phenolic compounds namely allylpyrocatechol and chavibetol, the main chemical compounds within the ethanolic extract of *P. betle*[16,17]. Three varieties of *P. betle* which are Kauri, Ghanagate and Bagerhati, are found to have higher potential than tea in preventing lipid peroxidation, and have the same antioxidant capacity as gallic acid [18]. Besides, essential oil of *P. betle* has a strong free radical scavenging activity and its activity is almost the same as ascorbic acid [19]. The compound allylpyrocatechol which is also found in the leaves has anti-inflammatory properties [20].

*Chlorella vulgaris* is a unicellular green alga [21] which can be found growing in fresh water [22]. Nutritional studies of *Chlorella* have revealed that this alga contains many intracellular phytochemicals namely carotenoids, chlorophyll, tocopherols, and ubiquinone; protein and others typical of green plants [23] besides flavonoid and polyphenol [24] which attributed to its antioxidant properties.

In Malaysia, palm oil is used as cooking oil. Tocotrienol-rich fraction (TRF) derived from palm oil consists of *α*-tocopherol and α-, β-, γ-, and δ-tocotrienol; all of which are isomers of vitamin E, and potent membrane-soluble antioxidants [25].

α-Tocopherol is an intracellular antioxidant which inhibits lipid peroxidation of polyunsaturated fatty acid located in lipid membrane [26]. Tocotrienol has been reported to have antioxidant property and suppressed reactive oxygen species production more efficiently than tocopherol [27], and showed promising non antioxidant activities in various *in vitro* and *in vivo* models [28].

In the present study, normal HDFs cells were used as our ageing model. Human diploid fibroblast (HDF) cells were sub cultured until passage 4, 15 and 30 which represent young, pre-senescent and senescent cells. The three age groups were treated with *P. betle*, *C. vulgaris* and TRF for 24 hours to evaluate the protective effect of these substances against cellular ageing, by measuring the activity of antioxidant enzymes.

## Methods

### P. betle and C. vulgaris extracts, and TRF

*P. betle* leaves were purchased from Ethnoresources Sdn. Bhd. (Sungai Buloh, Malaysia). The identification and voucher number (UKMB 29768) of the plant was obtained from Herbarium, Universiti Kebangsaan Malaysia, Bangi, Selangor, Malaysia. The extraction of *P. betle* was carried out as described by SO et al. [29]*.* Stock of *C. vulgaris* Beijerinck (strain 072) was obtained from University of Malaya Algae Culture Collection (UMACC, Kuala Lumpur, Malaysia). *C. vulgaris* was cultivated in the lab, and the hot water extraction of *C. vulgaris* was carried out as described by Saad *et al.*[30]. TRF Gold Tri E 50 which consists of 21.2% *α*-tocopherol and 78.9% tocotrienols was purchased from Sime Darby Bhd., Malaysia.

### Cell culture and the induction of senescence

This research has been approved by the Universiti Kebangsaan Malaysia Ethical Committee (Approval Project Code: FF-104-2007). The primary HDF was derived and maintained as described by Makpol *et al*. (2011) [31]. Primary HDFs were derived from the foreskins of three 9 to 12 year-old boys after circumcision. Written informed consents were obtained from parents of all subjects. The samples were aseptically collected and washed several times with 75% alcohol and phosphate buffered saline (PBS) containing 1% antibiotic-antimycotic solution (PAA, Austria). After removing the epidermis, the pure dermis was cut into small pieces and transferred into a falcon tube containing 0.03% collagenase type I solution (Worthington Biochemical Corporation, USA). Pure dermis was digested in the incubator shaker at 37°C for 6–12 h. Then, cells were rinsed with PBS before being cultured in Dulbecco Modified Eagle Medium (DMEM) containing 10% fetal bovine serum (FBS) (PAA, Austria) and 1% antibiotic-antimycotic solution at 37°C in 5% CO_2_ humidified incubator. After 5–6 days, the cultured HDFs were harvested by trypsinization and culture expand into new T25 culture flasks (Nunc, Denmark) with expansion degree of 1 : 4. When the subcultures reached 80–90% confluence, serial passaging was done by trypsinization and the number of population doublings (PDs) was monitored until HDFs reached senescence. The cells were used at passage 4 (young cells, population doubling; PD<12), passage 15 (pre-senescent cells, 30 < PD < 40), and passage 30 (Senescent cells, PD>55) in subsequent experiments. Young HDFs were incubated with 0.5, 0.4 and 0.3 mg/mL of TRF, hot water extract of *C. vulgaris* or aqueous extract of *P. betle* respectively, pre-senescent HDFs were incubated with 0.5, 0.2 or 0.3 mg/mL of TRF, hot water extract of *C. vulgaris* or aqueous extract of *P. betle* respectively while senescent HDFs were incubated with 0.5, 0.1 or 0.2 mg/mL of TRF, hot water extract of *C. vulgaris* or aqueous extract of *P. betle* respectively for 24 h. Untreated cells were cultured in DMEM containing 10% FBS (PAA, Austria). The media for the untreated cells were changed in parallel to the treated cells. The untreated and treated cells were harvested on the same day.

### Morphology analysis and senescence-associated β-galactosidase (SA β-gal) staining

The cells were divided into three experimental groups, based on their age: young, pre-senescent and senescent. Each group was treated with *P. betle* and *C. vulgaris* extracts, and TRF. SA β-gal activity, the molecular marker of HDF cellular ageing *in vitro*, was determined using a senescent cell staining kit (Sigma, USA) according to the manufacturer’s instructions. Cells (2 × 10^4^) cultured in a 6-well plate were washed twice with 1× PBS. Cells were then fixed with 1.5 mL of 1× fixation buffer and incubated at room temperature for 7 minutes. While waiting for incubation, staining mixture was prepared by mixing 1 mL of staining solution 10×, 125 μL of reagent B, 125 μL of reagent C, 250 μL of X-gal solution (pre-warmed for 1 h at 37°C) and 8.5 mL of miliQ water. After 7 minutes, cells were washed for three times with 1× PBS, followed by the addition of 1 mL of the staining mixture. Cells were incubated in a humidified incubator at 37°C for 4 h without CO_2_ supply. Blue stain was visible after 4 h of incubation. Cells were viewed with an inverted light microscope using 100× magnification. A total of 100 cells were observed in eight random fields, and the number of blue cells was counted. The percentage of blue-stained cells was calculated as the number of blue cells divided by the total of counted cells.

### Protein extraction

Following the 24-h treatments, HDFs (1 × 10^6^ cells) were trypsinised (0.25% trypsin, Hyclone, Australia) and harvested by centrifugation. The cell pellets were washed with 600 μL of cold PBS and incubated for 10 minutes in ice. The cell suspension was centrifuged at the maximum speed for 10 seconds at room temperature. The supernatant was discarded, while the pellets were stored at 4°C. A total of 200 μL of lysis buffer were mixed with the pellets and incubated on ice for 30 minutes. The suspension was centrifuged at the maximum speed (4°C for 30 minutes), where the supernatant was collected and stored at −80°C.

### Determination of total protein concentration

Total protein concentration was determined by using Bradford assay [32]. Briefly, 10 mg/mL of bovine serum albumin (Sigma, US) was prepared as standard protein solutions: 5, 10, 15, 20, 25, 30, and 35 μg/mL respectively. Then, 200 μL of Bradford reagent (Bio-Rad) was added to the standard, totalling to 800 μL as the final volume. Standards were prepared in duplicates, and the absorbance was measured at 595 nm using VersaMax tunable microplate reader (Molecular Devices, USA). Standard curve was plotted using the average of the duplicate. Concentrations of the samples were interpolated from the standard curve.

### Enzyme extraction

Following the 24-h treatments, HDFs (1 × 10^6^ cells) were trypsinised (0.25% trypsin, Hyclone, Australia) and harvested by centrifugation. One mL of PBS was used to wash the cells, followed by centrifugation at 600 rpm for 10 minutes, at 4°C. Then supernatant was discarded and the pellets were resuspended in 2 mL of 50 mM PBS. The cells were sonicated for 2 minutes at 4°C, followed by centrifugation at the maximum speed at 4°C. The supernatant was collected and stored at −80°C.

### Catalase activity assay

The activity of catalase was determined according to Aebi *et al.* (1984) [33]. A total of 700 μL of enzyme extract and 350 μL of 30 mM hydrogen peroxide (Sigma, US) were mixed. The absorbance of the mixture was determined at 240 nm 30 seconds later. Catalase activity was calculated by using the formula below:

Catalase Activity (mU/ mg protein) = (2.3) x –log (OD)/ Δ t × d.f × 1/[protein]

Remarks:

Δ t = time (seconds)

d.f = dilution factor (3×)

[protein] = protein concentration (mg/ml)

### Superoxide dismutase activity assay

Superoxide dismutase (SOD) activity was determined according to Beyer and Fridovich (1987) [34]. Substrate of SOD (NBT) was freshly prepared for every assay, by adding 1.5 mL of 30 mg/mL L-methionine (Sigma, USA), 1 mL of 14.1 mg/mL nitroblue tetrazolium (Sigma, USA), and 1 mL of 1% Triton X (Sigma, USA) to 27 mL of 50 mM PBS pH 7.8 with EDTA. One mL of SOD substrate was mixed with 20 μL of enzyme extract, followed by 10 μL of riboflavin (Sigma, USA). PBS was used as the negative control. Samples were incubated under 20 W light for 7 minutes, and the absorbance was measured at 560 nm. The specific activity of SOD was calculated by the formula below:

Percentage of inhibition = Absorbance (control-sample)/ Absorbance control × 100 × d.f

Unit enzyme = 1 unit inhibit 50% reduction rate of NBT

SOD specific activity (mU/mg protein) = Inhibition percentage (%)/ V × [protein] × t

Remarks:

d.f = dilution factor

V = enzyme volume (mL)

[protein] = protein concentration (mg/ mL)

t = time (minutes)

### Glutathione peroxidase activity assay

Glutathione peroxidase (GPx) activity was determined according to the method by Paglia and Valentine (1967) [35]. GPx substrate was freshly prepared by adding 20 μL of glutathione reductase (Sigma, USA), 5 mg reduced formed of glutathione (GSH) (Sigma, USA), 24 mg of reduced formed of nicotinamide adenine dinucleotide phosphate (NADPH) (Sigma, USA), and 25 mg of sodium azide (Hopkin & William, England) into 100 mL of 50 mM PBS pH 7.0 with EDTA.

2.88 mL of substrate was added with 20 μL enzyme extract in a cuvette. Then 100 μL of 2.2 mM of hydrogen peroxide (Sigma, US) was added. After 5 minutes, absorbance was measured at 340 nm. PBS was used as the negative control. GPx specific activity was calculated by using the formula below:

GP× specific activity (mU/ mg protein) = (Δ OD /min × V_k_ )/(6.22 mM^-1^ × 1 cm x V_s_ × [protein])

Remarks:

Δ OD = absorbance of sample – absorbance of control

6.22 mM^-1^ × 1 cm = molar extinction coefficient of NADPH at 340 nm with cuvette thickness 1 cm.

V_k_ = cuvette volume (ml)

V_s_ = enzyme volume (mL)

[protein] = protein concentration (mg/ mL)

### Statistical analysis

The data were statistically analysed by one-way ANOVA. P < 0.05 was considered significant.

## Results

### Cell morphology

**Figure 1:**
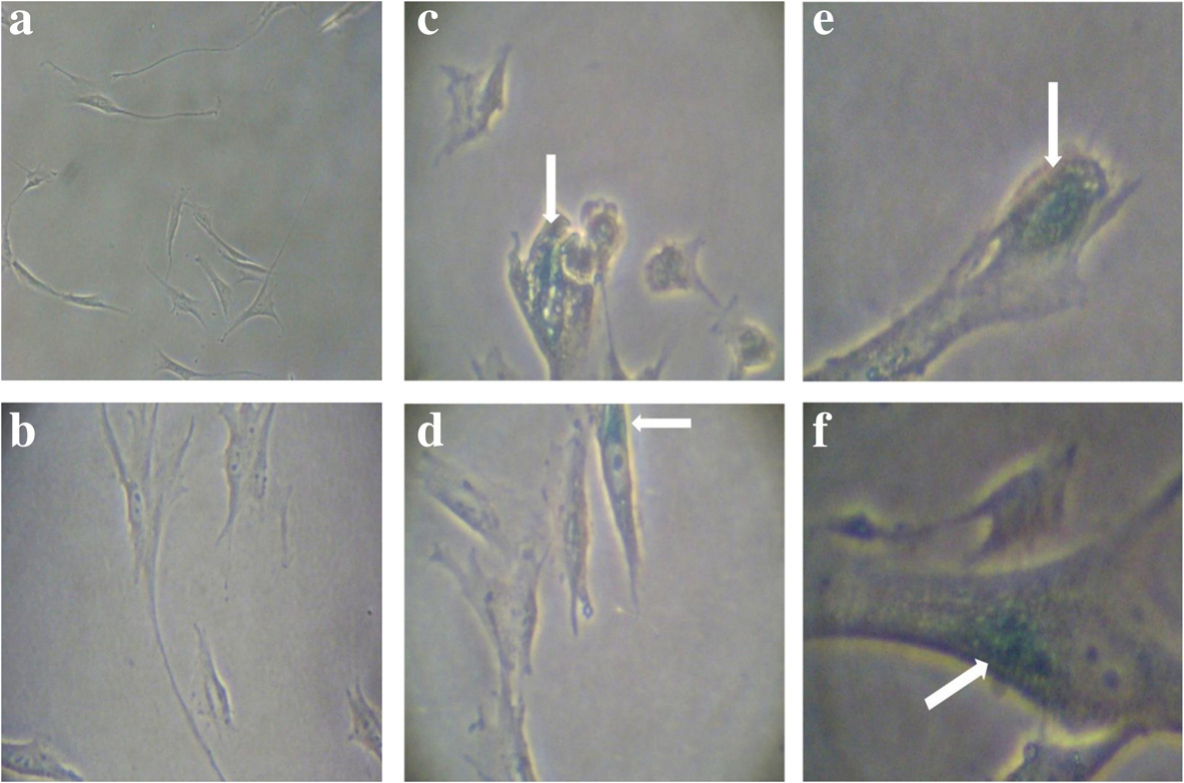
**Morphological changes of HDFs in culture.** Young **(a)**, pre-senescent **(b)** senescent HDFs **(c)**, senescent HDFs treated for 24 h with 0.2 mg/ml aqueous extract of *P. betle***(d)**, 0.1 mg/ml hot water extract of *C. vulgaris***(e)**, and 0.5 mg/ml TRF **(f)**. Cells expressing SA β-gal were stained blue (white arrows). Micrographs are shown at 100× magnification.

Morphological changes were observed with ageing of HDFs. Young HDFs displayed the normal spindle shape, common for fibroblasts (Figure 1a). With senescence, the cell volume and nucleus size of HDFs increased, and the cells became larger and flattened, while displaying increasing accumulation of vacuoles (Figure 1b-c). Morphologies of the senescent HDFs treated with the extracts and TRF were not affected (Figure 1d-f).

### SA β-gal expression

**Figure 2:**
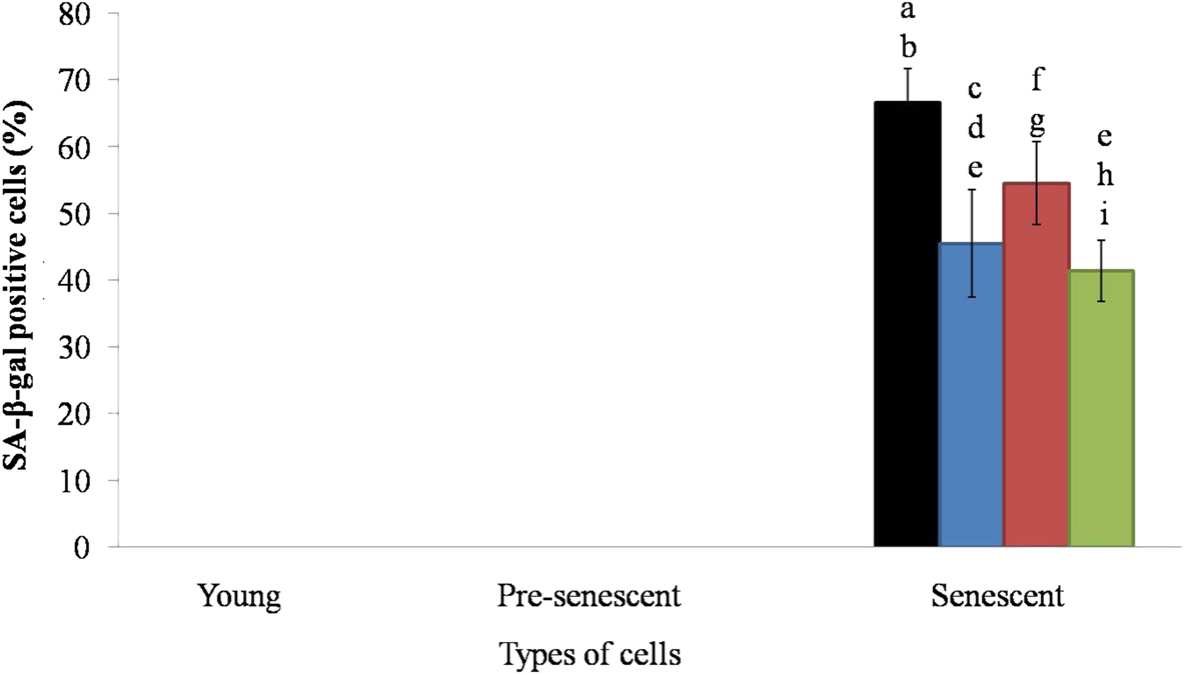
**Beta-galactosidase expressions in HDFs.** HDFs were treated for 24 h with 0.2 mg/ml aqueous extract of *P. betle*, 0.1 mg/ml hot water extract of *C. vulgaris* and 0.5 mg/ml TRF. Black, blue, red and green bars represent control, *P. betle*, *C. vulgaris*, and TRF treatments, respectively. ^a^p < 0.05 compared to young HDFs, ^b^p < 0.05 compared to pre-senescent HDFs, ^c^p < 0.05 compared to *P. betle*-treated young HDFs, ^d^p < 0.05 compared to *P. betle*-treated pre-senescent HDFs, ^e^p < 0.05 compared to senescent HDFs, ^f^p < 0.05 compared to *C. vulgaris*-treated young HDFs, ^g^p < 0.05 compared to *C. vulgaris*-treated pre-senescent HDFs, ^h^p < 0.05 compared to TRF-treated young HDFs, ^i^p < 0.05 compared to TRF-treated senescent HDFs. Data are presented as mean ± SEM, n = 6.

Among the three age groups of HDFs, the senescent cells displayed the highest percentage (70%) of blue-stained cells, while no blue-stained cells were observed in the other two groups (Figure 2). When the three groups were subjected to treatment with *P. betle* and *C. vulgaris* extracts and TRF, the treatments did not affect the young and pre-senescent HDFs, but the *P. betle* extract and TRF significantly reduced the number of blue-stained cells (45% and 40%, respectively). However, treatment with *C. vulgaris* extract produced no significant difference.

### Catalase enzyme activity

**Figure 3:**
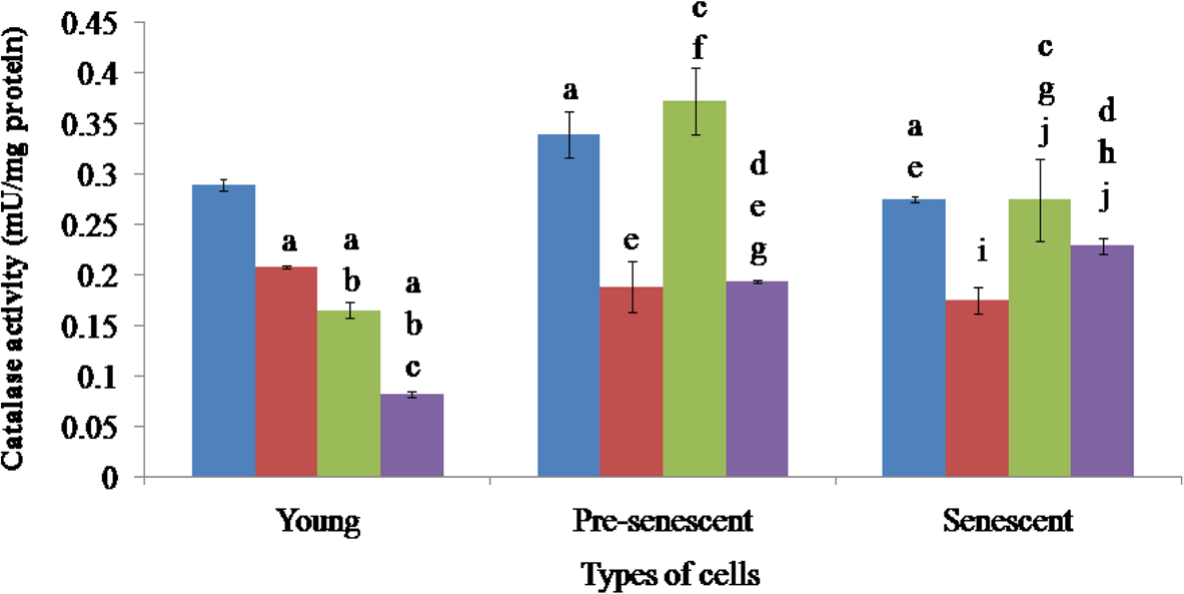
**Catalase activities of HDFs treated with *P. betle*, *C. vulgaris *and TRF.** Blue, red, green and violet bars represent control, *P. betle*, *C. vulgaris*, and TRF treatments, respectively. ^a^p < 0.05 compared to young HDFs, ^b^p < 0.05 compared to *P. betle*-treated young HDFs, ^c^p < 0.05 compared to *C. vulgaris*-treated young HDFs, ^d^p < 0.05 compared to TRF-treated young HDFs,^e^p < 0.05 compared to pre-senescent HDFs, ^f^p < 0.05 compared to *P. betle*-treated pre-senescent HDFs, ^g^p < 0.05 compared to *C. vulgaris*-treated pre-senescent HDFs, ^h^p < 0.05 compared to TRF-treated pre-senescent HDFs, ^i^p < 0.05 compared to senescent HDFs, ^j^p < 0.05 compared to *P. betle*-treated senescent HDFs. Data are presented as mean ± SEM, n = 6.

When treated with the extracts and TRF, the young cells displayed significantly reduced catalase activity (Figure 3). The biggest reduction was brought about by TRF, where the activity was reduced from 0.28 to 0.07 mU/mg protein, followed by *C. vulgaris* extract (0.17 mU/mg), and *P. betle* extract (0.21 mU/mg). The effects of the treatments were significantly different from each other in this particular age group.

Catalase activity in the pre-senescent cells (0.34 mU/mg) was significantly higher than that of the young cells, and was significantly decreased by half, by treatments with *P. betle* extract (0.17 mU/mg) and TRF (0.18 mU/mg), but not by the *C. vulgaris* extract (0.37 mU/mg). Treatments of the pre-senescent cells by *C. vulgaris* and TRF significantly produced different effects in the pre-senescent HDFs.

The senescent HDFs displayed a significantly lower catalase activity (0.26 mU/mg protein) than that of the young and pre-senescent cells. Treatment of the senescent cells with *P. betle* significantly reduced the catalase activity (0.16 mU/mg protein), but not with *C. vulgaris* and TRF. However, the treatment with *C. vulgaris* significantly increased and decreased the catalase activities in senescent HDFs as compared to the young and pre-senescent HDFs, respectively; and increased the activity compared to the treatment by *P. betle.* Compared to the other age groups, treatment by TRF in this age group produced a significantly higher activity. Treatment with TRF also produced a significantly higher catalase activity, in comparison to that by the *P. betle* extract.

### Superoxide dismutase enzyme (SOD) activity

**Figure 4:**
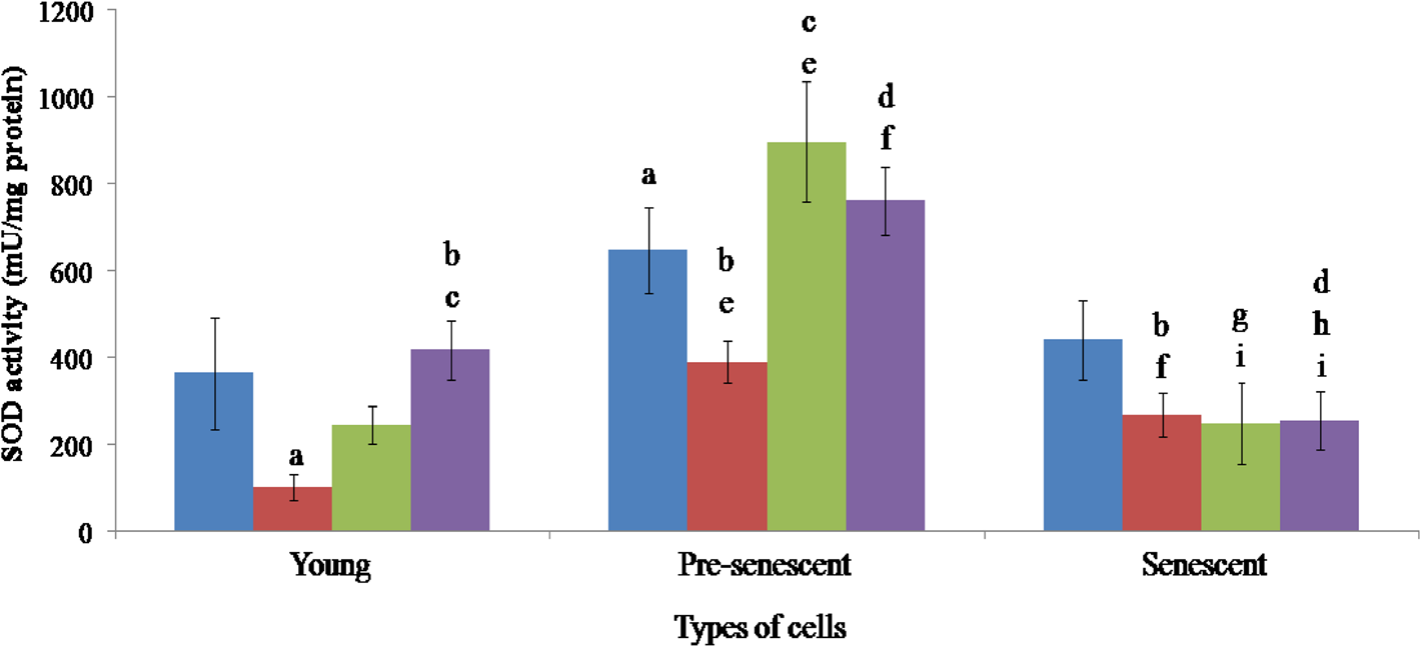
**SOD activities of HDFs treated with *P. betle*, *C. vulgaris *and TRF.** Blue, red, green and violet bars represent control, *P. betle*, *C. vulgaris*, and TRF treatments, respectively. ^a^p < 0.05 compared to young HDFs, ^b^p < 0.05 compared to *P. betle*-treated young HDFs, ^c^p < 0.05 compared to *C. vulgaris*-treated young HDFs, ^d^p < 0.05 compared to TRF-treated young HDFs, ^e^p < 0.05 compared to pre-senescent HDFs, ^f^p < 0.05 compared to *P. betle*-treated pre-senescent HDFs, ^g^p < 0.05 compared to *C. vulgaris*-treated pre-senescent HDFs, ^h^p < 0.05 compared to TRF-treated pre-senescent HDFs, ^i^p < 0.05 compared to senescent HDFs. Data are presented as mean ± SEM, n = 6.

The SOD activity was the highest in the pre-senescent (630 mU/mg protein), followed by the senescent (395 mU/mg protein), and the young HDFs (355 mU/mg protein, Figure 4).

The SOD activity in the young cells was significantly reduced following treatment with *P. betle* (100 mU/mg protein). Treatment with TRF (405 mU/mg protein) resulted in a significantly higher activity than the ones treated with *P. betle* and *C. vulgaris* (250 mU/mg protein).

Following treatment with *P. betle*, the activity in pre-senescent cells was significantly decreased (400 mU/mg protein), but significantly higher than that in the young. Treatment with *C. vulgaris* (875 mU/mg protein) and TRF (750 mU/mg protein) significantly increased the activity of SOD in pre-senescent HDFs as compared to young treated HDFs.

Treatment of the senescent cells (395 mU/ml protein) with *C. vulgaris* (250 mU/mg protein) and TRF (253 mU/mg protein) resulted in a significant reduction of SOD activity, even when compared to those in the pre-senescent cells. TRF treatment produced significantly lowered SOD activity as compared to young and pre-senescent TRF treated groups. *P. betle* treatment only produced significant effect when compared to the other age groups.

### Glutathione peroxidase enzyme activity

**Figure 5:**
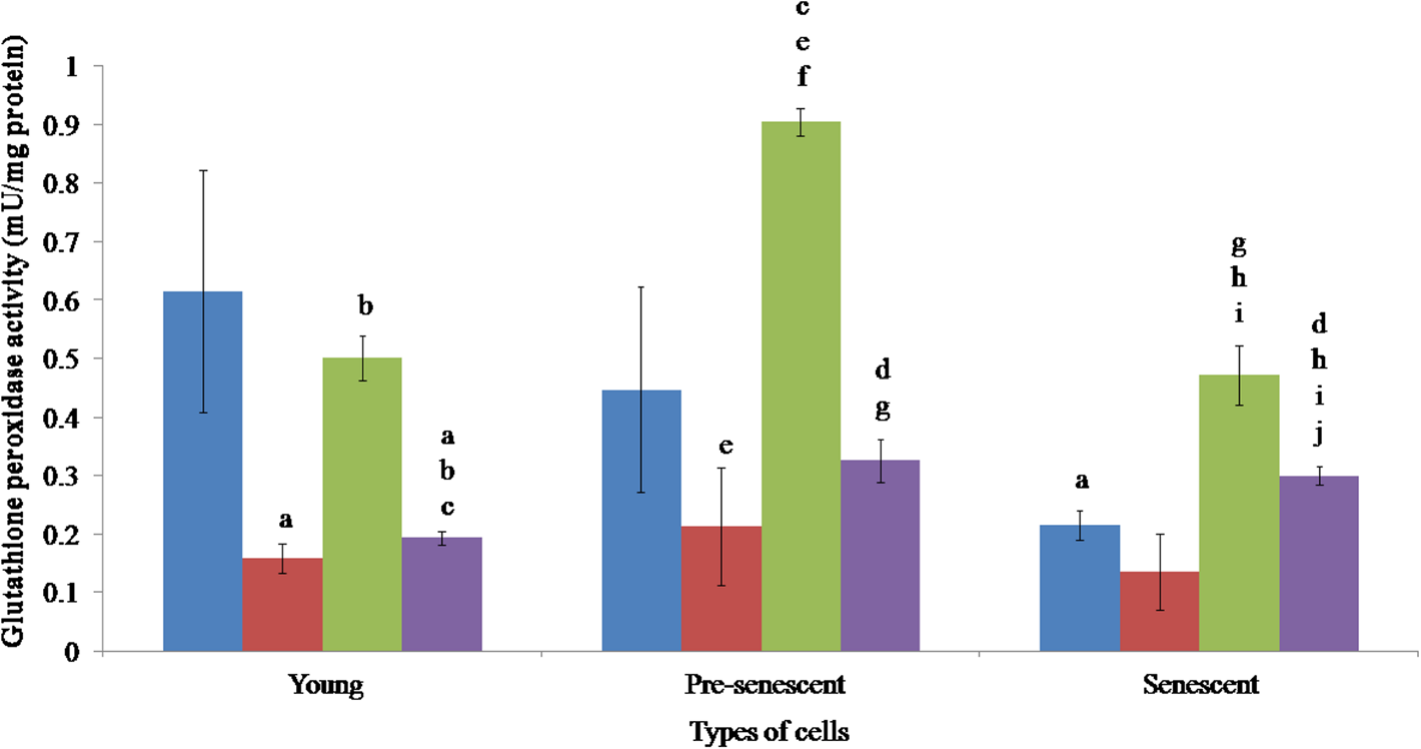
**Glutathione peroxidase activities of HDFs treated with *P. betle *, *C. vulgaris *and TRF.** Blue, red, green and violet bars represent control, *P. betle*, *C. vulgaris*, and TRF treatments, respectively. ^a^p < 0.05 compared to young HDFs, ^b^p < 0.05 compared to *P. betle* -treated young HDFs, ^c^p < 0.05 compared to *C. vulgaris*-treated young HDFs,^d^p < 0.05 compared to TRF-treated young HDFs, ^e^p < 0.05 compared to pre-senescent HDFs, ^f^p < 0.05 compared to *P. betle*-treated pre-senescent HDFs, ^g^p < 0.05 compared to *C. vulgaris*-treated pre-senescent HDFs, ^h^p < 0.05 compared to senescent HDFs, ^i^p < 0.05 compared to *P. betle*-treated senescent HDFs, ^j^p < 0.05 compared to *C. vulgaris*-treated pre-senescent HDFs. Data are presented as mean ± SEM, n = 6.

In young HDFs (Figure 5), the activity of GPx (0.61 mU/mg protein) was significantly reduced by treatment with *P. betle* (0.16 mU/mg protein) and TRF (0.20 mU/mg protein). Treatment with *C. vulgaris* (0.50 mU/mg protein) did not lower the enzyme activity, which was significantly produced a higher enzyme activity than the *P. betle* treatment. Treatment with TRF produced a significant effect when compared to the other two extracts.

The activity of GPx in the pre-senescent cells (0.46 mU/mg protein) was significantly affected by all the treatments. *P. betle* extract (0.24 mU/mg protein) and TRF (0.34 mU/mg protein) significantly reduced the enzyme activity, but *C. vulgaris* (0.91 mU/mg protein) produced the opposite effect, which was also significantly higher than that in the young cells.

The activity of GPx in the senescent cells (0.25 mU/mg protein) was significantly lower than that of the young HDFs. The enzyme activity was significantly increased when the senescent cells were treated with *C. vulgaris* (0.48 mU/mg protein) and TRF (0.32 mU/mg protein), but no significant effect was observed from *P. betle* treatment (0.16 mU/mg protein). The effect of the *C. vulgaris* treatment was significantly lower than that observed in the pre-senescent cells, but higher than the effect produced by *P. betle* treatment in the pre-senescence. The effect resulting from the TRF treatment was also significantly different than that produced by *P. betle* and *C. vulgaris* extracts in the same age group.

## Discussion

The present study is focused on comparing the effect of *P. betle*, *C. vulgaris* and TRF on cellular ageing in HDFs. The morphology of the HDFs was shown to change with age, similar to previous studies that showed senescing cells undergo morphological changes to become flattened and enlarged in size [31,36]. The size increment was largely due to the increasing number and mass of mitochondria and autophagic vacuoles, and the accumulation of nuclear proteins and other metabolites [5]. Mitochondria provide cellular energy in the form of ATP, but the accumulating oxidative damage during ageing lowers the function of mitochondria [37]. In turn, the size and number of mitochondria was increased to counteract the decreasing biogenesis ability of mitochondria during ageing. Accumulation of nuclear protein caused the expansion of the nucleus, contributing to the enlargement of the cells [38], while the rise in the number of auto lysosomal enzymes contributes to enlargement of vacuoles [39]. Ageing is an irreversible process [5,11]. However, treatment of the senescent HDFs with the *P.betle* and *C. vulgaris* extracts and TRF reduced the number of SA β-gal positive cells, suggesting a reversal in cellular ageing is possible [31].

SA β-gal is a common *in vitro* ageing marker, which has been observed in senescent human diploid fibroblasts (HDFs), skin, liver, muscle, and endothelial cells [40]. Our result showed that only senescent (passage 30) cells displayed blue-stained cells. Increased activity of β-galactosidase (a lysosomal enzyme) is associated to auto lysosomal activity, which may cause cells to undergo ageing and finally death [39].

Our study demonstrated that *P. betle* and TRF possessed anti-ageing effect, based on the reduced expression of SA β-gal, in senescent HDFs. *P. betle* contains phenolic components such as kavibetol and alylpirocatechol [16]. The antioxidant actions of these components could reduce oxidative damage that occurred during ageing. Modulation of oxidative stress can directly control ageing [41]. Telomere shortening and accumulation of DNA damage are the mechanisms that force cells to undergo ageing. α-Tocopherol and γ-tocotrienol have been reported to have protective effect against telomere shortening by increasing telomerase activity and protect DNA damage induced by hydrogen peroxide [31,42,43]. As an antioxidant, TRF reduces oxidative stress and low density lipoprotein peroxidation [44], by donating electron in the form of phenolic hydrogen to the lipid radical [45]. TRF also provide protective effect towards Alzheimer disease, which is caused by oxidative damage [46].

Relatively, *C. vulgaris* exhibited an anti-ageing effect to a lesser extent. Although the number of positive SA β-gal senescent cells were reduced, but the effect was not a significant one. This trend was similar with an earlier study where there was no significant reduction in DNA oxidative damage in *C. vulgaris*-treated leukocyte [47].

Manifestation of ageing is accompanied by the involvement of antioxidant molecules. Antioxidant enzymes such as catalase, SOD and GPx play a major role in counteracting the effect of oxidative stress, which is a factor of ageing [6,48]. These enzymes are often targeted in anti-ageing and drug discovery research [48,49]. Furthermore, a study by Remmen et al. (2004) showed that mice lacking both SOD and GPx genes were more sensitive towards oxidative stress [50].

Our results showed that in ageing HDFs, catalase and GPx activities were decreased, while SOD activity was at its peak during pre-senescent stage. SOD level has been shown to be age-independent [51]. These effects were also observed in human fibroblasts under oxidative stress induced by ultraviolet-A irradiation and 8-metocypsoralen [49].

As ageing progresses, increased SOD activity resulted in a high level of hydrogen peroxide, but reduced catalase and GPx activities have caused the accumulation of hydrogen peroxide [49]. GPx reduces hydrogen peroxide by oxidizing GSH, and catalase converts hydrogen peroxide to water and oxygen [8,13]. Thus, the increasing SOD activity, and decreasing GPx and catalase activities during ageing damaged the antioxidant enzyme homeostasis within a cells and forced it to undergo ageing.

Compounds like chavibetol and alylpirocatechol from *P. betle*[16,17,52] are believed to be able to prevent degenerative diseases [53]. This study demonstrated that treatment of young, pre-senescent and senescent HDFs with aqueous extract of *P. betle* reduced the activity of catalase. The same effect was observed in rats that received *P. betle* ethanolic extract as a pre treatment, later induced with cadmium chloride to induce oxidative stress [48]. The reduction in catalase catalytic activity could be attributed to the reduced amount of H_2_O_2_ available, by the ability of *P. betle* to reduce oxidative stress in fibroblast cells by non-enzymatic antioxidant activity [54]. The activity of *P. betle* was supported by a discovery made by Dasgupta and De (2004), which proved that aqueous extract of *P. betle* leaves reduced non-enzymatic lipid peroxidation by free radical scavenging effect [18]. The *P. betle* extract also reduced SOD activities in young and pre-senescent HDFs, which is similar to a study done by Prabu et al. (2012). In their study, rats were induced with cadmium chloride to induce oxidative stress. Rats that received *P. betle* ethanolic extract as a pre treatment, showed a reduced SOD activity [48].

In this study, *C. vulgaris* hot water extract significantly reduced and increased the activities of SOD and GPx, respectively in senescent HDFs. *C. vulgaris* contains polyphenol and flavonoid that portray antioxidant activity by free radical scavenging. Extracts isolated from *C. vulgaris* posses redox properties, which are vital in absorbing and neutralizing free radicals, quenching ROS, and decomposing peroxides [24]. The potent antioxidant activity displayed by flavonoids owes to the hydroxyl group substituent on the flavonoid nucleus [55]. Non-enzymatic antioxidant activity of *C. vulgaris* might have reduced superoxides, hence the reduction in SOD activity, which may be attributed to the down-regulation of SOD expression. This resulted in a net increase in hydrogen peroxide level, which then brought about the increase in GPx activity [49].

TRF from palm oil consists of 75% tocotrienol and 25% tocopherol [44]. Tocopherol is a good cellular antioxidant due to its ability to stop the peroxidation of polyunsaturated fatty acid at the biological membrane [26], by removing lipid radical peroxyl [56]. A study by Halliwell & Gutteridge (2002) [57] also showed that lipid radical peroxyl is more susceptible to react with TRF, compared to lipids in the membrane, due to the presence of unsaturated side chain [27]. Choi and Lee (2009) also demonstrated that TRF is more effective than the other fractions they produced, in neutralizing free radicals and lipid radical peroxyl and destroying prooxidant metal [26]. This study has shown that TRF effectively reduced SOD activity, but raised glutathione peroxidise activity, which was the same effect achieved by *C. vulgaris* treatment.

The present study demonstrated that during ageing, HDFs treated with *C. vulgaris* exhibited the highest antioxidant enzyme activities, compared to those treated with *P. betle* and TRF. *C. vulgari*s contains lowest antioxidants compared to *P. betle* and TRF. Hence, fibroblasts treated with *C. vulgaris* had to respond with increasing antioxidant enzyme activities to maintain the redox balance [11]. This outcome was similar with a study showing that SOD and GPx increased in heart allograph with increased oxidative damage [58].

Furthermore, previous study showed that total content polyphenol of *C. vulgaris* was 1.34×10-2 mg galic acid/mg [24]. On the other hand, total content polyphenol of *P. betle* variety Kauri was 0.96 mg galic acid/mg. Furthermore, TRF consists of 85.6% inhibition in DPPH antioxidant assay compared to quercetin [59]. Relatively, *C. vulgaris* contained less antioxidants compared to *P. betle* and TRF.

## Conclusion

In this study, the level of SA β-gal of HDFs increased with age. Aqueous extract of *P. betle* and TRF were able to substantially delay the ageing of fibroblast cells, while *C. vulgaris* generated a weaker outcome. *P. betle* showed the strongest antioxidant activity by reducing SA β-gal expression, catalase activities in all age groups, and SOD activity. TRF exhibited a strong antioxidant activity by reducing SA β-gal expression, and SOD activity in senescent HDFs. *C. vulgaris* extract managed to reduce SOD activity in senescent HDFs. Nevertheless, this study requires further investigation in order to elucidate the exact mechanism of antioxidant activities of the extracts and TRF.

## Abbreviations

HDFs: Human diploid fibroblasts; TRF: Tocotrienol-rich fraction; SOD: Superoxide dismutase; SA β-gal: Senescence-associated β-galactosidase; GPx: Glutathione peroxidase.

## Competing interests

The authors declare that they have no competing interests.

## Authors’ contributions

SM was the Principal Investigator who designed the study and revised the manuscript. TWY carried out the lab work and drafted the manuscript. FACR, KTA and YAMY were involved in data acquisition and revising the manuscript. All authors read and approved the final manuscript.

## Pre-publication history

The pre-publication history for this paper can be accessed here:

http://www.biomedcentral.com/1472-6882/13/210/prepub
